# Contrast-Enhanced Ultrasound-Guided Radiofrequency Ablation of Renal Tumors

**DOI:** 10.15586/jkcvhl.2018.100

**Published:** 2018-02-02

**Authors:** Dan O’Neal, Tal Cohen, Cynthia Peterson, Richard G. Barr

**Affiliations:** 1 Department of Radiology, Northeastern Ohio Medical University, Rootstown, Ohio, USA; 2 Kent State University, Salem, OH, USA; 3 Ultrasound Training, Southwoods Imaging, Youngstown, OH, USA

**Keywords:** contrast-enhanced ultrasound, radiofrequency ablation, renal tumor ablation, small renal masses, ultrasound contrast agents

## Abstract

Although only limited long-term studies evaluating thermal ablation of renal masses have been performed, it appears that thermal ablation has a comparable 5-year success rate to that of partial or total nephrectomy. This technique is often used in patients who are not good candidates for partial or total nephrectomy. Contrast-enhanced ultrasound (CEUS) has been recently approved by the Food and Drug Administration for characterization of focal liver lesions in adults and pediatric patients. CEUS can be used off label for renal applications and has been used for years in Europe and Asia. It has several advantages over contrast-enhanced computed tomography for use as the technique to guide and evaluate efficacy of thermal ablation of renal masses. These include the ability to visualize small amounts of enhancement, repeat dosing to evaluate efficacy of an ablation during a procedure, thin slice thickness, and real-time visualization. Ultrasound contrast is also non-nephrotoxic and non-hepatotoxic, allowing evaluation of patients with renal insufficiency. This article reviews the use of CEUS for the guidance and follow-up of thermal ablative procedures of renal masses.

## Introduction

According to the American Cancer Society, in the United States, 63,340 new cases of renal cancer will occur in 2018, an incidence that has been increasing since the 1990s and has only recently begun to level off (1). Much of this increase has been attributed to the improved diagnosis of small renal masses (SRM) that are localized to the kidney and often incidentally found in asymptomatic patients being imaged for other reasons (1). The most common type of renal cancer found is renal cell carcinoma (RCC), which accounts for 9 out of every 10 cases and makes up 3% of all adult neoplasms (1, 2). When examining cases of RCC, Bhan et al. (3) found that 48–66% of these tumors can be attributed to these incidentally discovered SRM. Atri et al. (2) further state that the greatest increase in RCC diagnosis has been early stage T1 tumors, which they define as SRM when the tumor is less than 4 cm in axial diameter, and has an observed 5-year survival rate of 81%. Stages II and III tumors have an observed survival rate of 74% and 53%, respectively, while the survival rate for stage IV tumors is as low as 8% (1). It is important to note, however, that these data include patients with RCC who may have died of other causes. With this relatively recent rise in incidence, along with the ever-advancing technology in the medical field, treatment of these renal cancers, especially RCC, has become a frequently discussed topic.

The long time tried and true method for treating RCC is surgical resection, which carries a low mortality and high success rate (1). For this reason, it should remain the standard of care for patients with potentially curable RCC. With that being said, a significant number of patients who have renal cancer are not favorable surgical candidates. There are a variety of reasons that may make a patient ineligible for partial nephrectomy or surgical resection. These include significant comorbidities, a life expectancy between 1 and 10 years, chronic renal failure, patient only having one or a transplant kidney, advanced age, bilateral RCC, Von Hippel–Lindau (VHL) disease, or refusal of conventional therapy (4–6). Many of these contraindications can be commonly found in the elderly, which is problematic since the average age of diagnosis of RCC is 64 (1, 7). Chronic renal failure is especially important because those requiring dialysis also have an increased risk of developing RCC (1). Radiofrequency ablation (RFA) or other forms of thermal ablation are possible alternative treatments for many of these patients.

Though RFA is a relatively new treatment for renal tumors and long-term efficacy remains to be seen, the initial data suggest that not only is it safe and well tolerated by patients, but also has a comparable 5-year success rate to that of partial or total nephrectomy (4, 5, 8). One of its biggest benefits is that the procedure is renal sparing, and RFA is currently used for small tumors, usually less than 5 cm, in patients who are not candidates for surgical removal (7). Besides being used as an attempted cure of RCC, it may also be used to treat RCC-associated intractable hematuria, local recurrences, isolated metastases, and palliation of symptoms (6). RFA is considered a minimally invasive procedure in which a probe is inserted into the target tissue and alternating electrical currents are used to generate heat to destroy tumor cells and associated neovascularity. This procedure is guided by advanced imaging techniques that help position the probe and are paramount in evaluating the success of ablation (9). Traditionally, computed tomography (CT) is used in the initial pre-ablation evaluation, as well as to guide the probe placement, evaluate the ablation zone, and tumor post-ablation. Ultrasound (US) has also been used in combination with CT or alone because of its ability to provide a real-time image. Both these techniques, however, have some limitations in the form of contrast-related problems (e.g., renal insufficiency), lack of live image in CT, and reduced visualization and accuracy in US. One possible alternative to guide the RFA procedure is to use contrast-enhanced ultrasound (CEUS), an advanced technique that utilizes ultrasound contrast agents (UCAs) to improve lesion visualization in difficult cases and to either immediately or at a later time detect residual tumor after ablation (4, 10).

## Ultrasound Contrast Agents

UCAs are gas-containing microspheres with an outer shell of lipid, protein or polymer (11). With a diameter ranging from 1 to 10 μm, these microbubbles are roughly the size of a red blood cell. This size allows them to pass through capillaries and be delivered to any tissue that maintains circulation, all the while avoiding extravascular passage (8, 11, 12). With no extravascular passage, UCAs are considered to be pure blood pool agents that are neither filtered nor excreted by the renal system (4). However, the most important property of these agents is that the microspheres will resonate when exposed to frequencies that are commonly used in diagnostic ultrasonography (8, 9, 11). The resulting reflection and scatter from the resonating microspheres leads to increased echogenicity, allowing for real-time imaging of the microcirculation. The microspheres will last for about 5–7 min inside the blood vessels until they dissolve (11). The internal gas is then exhaled by the lungs while the shell is metabolized by the body.

In practice, UCAs possess a low incidence of side effects and are considered safe for patients, especially those with decreased renal function (4, 10, 12, 13). These patients benefit from the fact that UCAs are not excreted into the urine and therefore not nephrotoxic, a contradistinction to the agents used for CT and magnetic resonance imaging (MRI). They also have a very low rate, less than 0.002%, of anaphylactoid reactions, which is lower than that of CT contrast agents (10, 11). A previous reaction to CT contrast agents also does not preclude one from UCAs because the two agents are completely different. Currently, the Food and Drug Administration (FDA) (14) has approved the intravenous use of several UCAs for use in echocardiography, as well as one agent for use in hepatic imaging. However, many physicians around the world have found success utilizing this technique for imaging a variety of tissues, including renal tumors (10).

## CEUS of Renal Masses

Currently, complex renal cysts are evaluated using CT and classified by an interpretation standard, such as the Bosniak Classification System. This system ranges from I to IV, with category I having qualities of a simple cyst and category IV that of a clearly malignant tumor (12, 15). Category IIF is composed of more complex cystic lesions that need to be followed up because they cannot be classified as category II. Category III is reserved for those indeterminate cystic renal masses that are neither definitively benign nor malignant. These categories are then used to make decisions on whether surgical versus conservative management is best for the patient. In practice, however, there exists inter-reader variation in making distinction between categories II, IF, and III lesions, which poses potential problems in making these recommendations (12). It is with these types of tumors that CEUS has a significant advantage over CT.

The microspheres utilized in CEUS allow for real-time imaging of renal masses with improved depiction of renal vessels, solid tumor vascularity, and detection of septal and peripheral wall vascularity of complex renal masses (11, 12, 16). Enhancement with UCAs usually lasts for 5–7 min (11). The first to enhance is the arterial pedicle and the main branches, followed by the renal cortex a few seconds later (10, 11). With signals independent of the angle of insonation, the microspheres even provide adequate depiction of renal pole perfusions, something lacking in Doppler US (10). The next to follow is medullary perfusion, with the outer medulla enhancing before the gradually filling pyramids (10, 11). Many experts have also noticed that patients with chronic renal disease have less intense enhancement that fades earlier. As previously stated, CEUS is safe in these patients, unlike CT contrast, because it is not excreted into the urine and therefore not nephrotoxic. Historically, a common alternative to CT with contrast for patients with chronic renal disease is MRI with contrast. However, unlike MRI, CEUS can be used in patients with arthroprosthesis or pacemakers, and faces less of a problem of movement artifacts from patients who cannot stay still (9). Furthermore, there has been mounting concern about the use of gadolinium MRI contrast agents in patients with chronic renal failure and how it may be associated with nephrogenic systemic sclerosis (6, 9).

When using CEUS, a renal tumor is best depicted during the arterial phase, usually beginning about 15–20 s after contrast injection (9, 12). Meloni et al. (9) stated that during this phase, the hypervascularity of renal tumors will appear with brighter intensity than the surrounding normal renal parenchyma. They also noted that tumors had faster washout, leading to a hypoechoic appearance 30–40 s after contrast injection. Quaia et al. (12) and other researchers were able to use these characteristics-enhancing patterns in order to create vascularity profiles that can be used to determine the likelihood of a tumor being benign or malignant, similar to how the Bosniak classifications are used for CT (17). With regard to accuracy, several studies have shown CEUS to have comparable to improved results versus CT in the discovery and evaluation of complex renal cysts (5, 9, 12, 18). Quaia et al. (12) did note, however, that inter-reader agreement improved with increasing experience, indicating that one must consider the learning curve of using CEUS. With such encouraging results, less contraindications and cheaper cost versus CT and MRI, CEUS may not only become increasingly utilized in the evaluation and follow-up of complex renal cysts, but can also be applied to interventions such as RFA.

## CEUS-Guided RFA

Apart from initial tumor evaluation, CEUS has also shown promise in the localization, guidance and postoperative imaging with regard to RFA. RFA has found an increasing role in the treatment of small renal tumors in patients who are not candidates for surgical resection, as previously outlined in the Introduction (19). It is an outpatient procedure in which a probe is inserted into the tumor and delivers thermal energy within the tissue that is created via a high-frequency alternating current released from the active electrode at the tip (1, 7, 20). According to Boss et al. (7), the subsequent thermal damage will lead to coagulative necrosis and cellular death once the target temperature exceeds 48–50°C. The size of this area of necrosis is linearly dependent on treatment time and exponentially dependent on the temperature and can be predicted using biophysical relationships (7). However, the tissue destruction around the applicator tip limits the achievable ablation size by decreasing the energy deposition through increased electrical impedance. With this in mind, it is suggested for larger tumors (>2 cm) to use either multiple probes simultaneously or a single probe repositioned after each ablation to ensure complete destruction (4, 7, 9).

Traditionally, the RFA probes are placed under CT guidance. CT provides excellent visibility of the probe, but takes multiple steps of image capture and probe adjustment because it does not provide a live image (1, 8). In comparison, recent studies have shown that with its continuous image and vascular characterization, CEUS can allow for improved orientation and guidance of the probe into the target tumor (1, 10, 11). CEUS may then be used to evaluate the target zone after waiting for 5–10 min post-ablation to allow for the heat-generated gas to dissipate (10, 11). This helps eliminate artifact in the post-procedure image when evaluating the ablation zone. Ablation success is characterized as non-enhancement in the area of the previously enhancing lesion, while residual tumor will appear as a crescent-like enhancing region within the ablation zone (4, 9–11, 13). However, these post-treatment imaging results should be closely compared with pre-treatment imaging results so that one does not misinterpret larger blood vessels surrounding the region as residual tumor (10, 11, 13). Such timely postoperative imaging is not always possible when using CT because of the nephrotoxicity limitations associated with CT contrast agents. Furthermore, if residual tumor exists, it would take a separate procedure on a later date to fully treat (4, 5, 7). When using CEUS, this same residual tumor can be recognized shortly after the first ablation and targeted by repositioning the probe because US contrast can be safely administered multiple times per session.

With the probability of tumor recurrence after RFA to be estimated as high as 12.9% at 24 months, posttreatment follow-up imaging is critical (3). This is especially true when considering the findings of Sanchez (5) that most incomplete treatments using RFA are detected within the first 3 months. Traditionally, follow-up imaging for post-RFA of renal tumors utilizes either CT or MRI. However, there have been several recent studies showing the efficacy of CEUS in follow-up and how it is a reproducible technique that may be performed early in the follow-up because it carries no renal toxicity (6). Kong et al. (4) performed RFA on 63 patients with 64 RCCs and compared the results of CEUS with CT at 1-month follow-up imaging. They found that the concordance between the two modalities for detecting residual vascular enhancement was 100%, with CEUS having a sensitivity of 100% and a specificity of 96.6%. These findings are similar to those referenced in European Federation of Societies for Ultrasound in Medicine and Biology (EFSUMB) Guidelines and Recommendations on the Clinical Practice of Contrast Enhanced Ultrasound (10), which state that CEUS studies provided similar overall accuracy to that of CT/MRI in confirming the accuracy of treatment. Kong et al. (4) also noted that CEUS exhibits higher concordance with the definitive necrosis area than conventional US. The fact that CEUS has higher concordance with the area of necrosis is confirmed in the study of Johnson et al. (8) where they performed post-RFA imaging immediately after ablation and at 1 week on several pig kidneys and then compared the imaging data to the gross RFA lesions after dissection. However, not all studies have been as favorable to the use of CEUS over the traditional methods of CT/MRI.

A study by Hoeffel et al. (6) compared the results of CEUS with that of CT/MRI for 43 patients (66 renal tumors) post-RFA at 24 h and 6 weeks while using CT/MRI at 1 year as a reference standard. The study found that all imaging modalities were more accurate at 6 weeks than at 24 h and that CEUS had high specificity and predictive values for residual tumor detection (6). However, CEUS had lower sensitivity than that of CT/MRI, and had worse image quality at the upper renal poles and at the periphery of the treated areas. Hoeffel et al. also noted that CEUS performed better in the assessment of hypervascular tumors, a concept that has been noted in several other studies and could be related to the ability of UCAs to image the microvasculature (6, 9, 12). Other limitations of CEUS may include lesion location as well as the specific properties of UCAs when used in the kidneys. The intensity of the reflected echoes decreased significantly with depth, which means that deep lesions, or those in obese patients, may appear to have reduced vasculature (11). Bowel gas interposition may also interfere with the quality of CEUS evaluation, but Meloni et al. (9) stated that these limitations can be reduced with an imaging approach that minimizes depth and gas interference, as well as newer contrast-specific modes such as contrast pulse sequence imaging. CEUS has the advantage of no renal excretion so that the dense contrast in the collecting system does not mask an abnormality (11).

## CEUS Guidelines

The following outlines our standard procedure for RFA of renal masses under CEUS guidance:

(1) First a patient must be determined to be a candidate for RFA based on the pre-procedure imaging of the renal mass. Tumor size, position and stage, as well as other patient factors are used to determine candidacy. If the patient is a candidate, informed consent is obtained, and a pre-procedure physical examination and laboratory studies including standard coagulation profile are obtained.
(2) Either on the day of or a few days before the procedure, a pre-ablation CEUS is performed to evaluate the size and vascular supply of the tumor. These images can be used to plan the puncture site and needle trajectory, and will also be compared to the post-ablation images to determine success.
(3) Prior to the procedure, most patients will undergo conscious sedation, although general anesthesia may be required depending on certain patient factors and wishes. Once the patient is prepped and ready, the RFA needle is positioned within the mass using image guidance. Depending on the size or shape of the tumor, multiple probes may be placed before beginning ablation. Ablation is then performed for about 10–15 min depending on tumor size.
(4) After waiting 5 to 10 min for the heat-generated gas to dissipate, a repeat CEUS is performed to look for residual tumor. These images are compared closely to the pre-ablation CEUS images and if any residual tumor is detected, the RFA needle is repositioned for a repeat ablation. Once no residual tumor is viewed on post-ablation CEUS, the RFA probe is retracted slowly, while turned on, to ablate the track and reduce the risk of tumor seeding.
(5) Most studies follow a schedule of follow-up imaging every 3 months in the first year and every 6 months in the second year. Some choose to stagger CEUS and CT follow-up imaging over the first year, while others perform both at every follow-up. The first year is the most important because that is when most residual tumors are detected.

## Case Studies of CEUS RFA

The following case studies provide an overview of how to perform CEUS for guidance of RFA for renal tumors.

**Figure 1.**
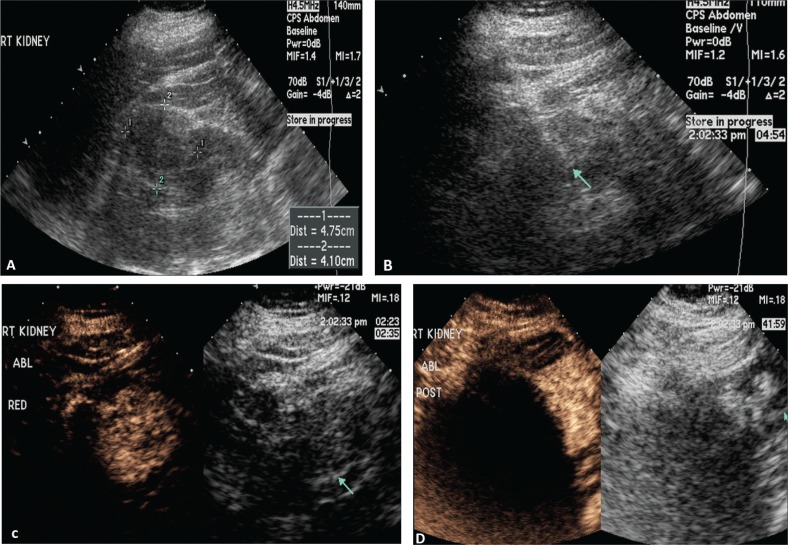
**Images demonstrating RFA of an RCC.** (A) B-mode image of the RCC (calipers) pre-procedure, (B) B-mode image demonstrating positioning of the RFA needle (arrow) in the RCC, (C) image after injection of ultrasound contrast confirming the needle (arrow on low MI B-mode image on right) within the tumor, and (D) CEUS post-procedure demonstrating complete ablation of the RCC (arrow, right).

Figure 1 demonstrates the steps in performing a CEUS RFA. Figure 1A is the unenhanced B-mode US before the procedure. In this case, the mass is easily identified. If the mass is not visualized well, a CEUS can be performed to confirm the size and location of the mass. Figure 1B shows the position of the RFA needle in the mass. Figure 1C is the image after the injection of the ultrasound contrast confirming the position of the needle in the mass. Figure 1D shows that there is no flow remaining in the renal mass, confirming complete ablation of the lesion.

**Figure 2.**
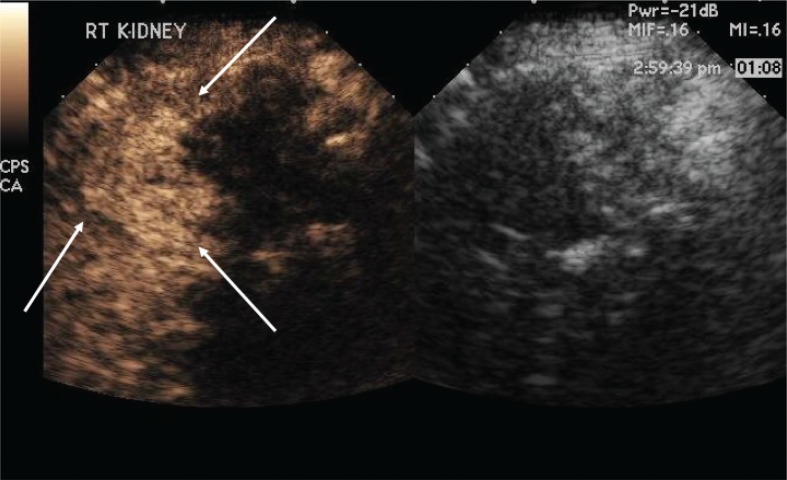
**Multiple doses of ultrasound contrast can be administered during the RFA procedure.** The contrast agents last for about 5 min so that residual enhancement is not present on additional doses. In this case, a CEUS study was performed after the first ablation. Note that there is residual tumor (arrows). The RFA needle can then be repositioned into the residual tumor for additional ablation. This confirms that the tumor is completely ablated at the first setting. The similar process cannot be performed in CT because only one dose of CT contrast can be administered.

Figure 2 demonstrates that after the first ablation, a second dose of ultrasound contrast can be performed to evaluate for residual tumor. The CEUS contrast lasts for about 5 min. During the RFA, gas bubbles are generated and appear as CEUS contrast when imaging. Waiting for about 5 min after the completion of the ablation will eliminate this problem. If bubbles still remain in real-time CEUS imaging, the bubbles generated from the procedure will not move whereas the UCA bubbles in residual tumor will be visualized as moving. In this case, a large area of residual tumor (arrows) remains. The CEUS image can be used to reposition the RFA needle into the residual tumor.

**Figure 3.**
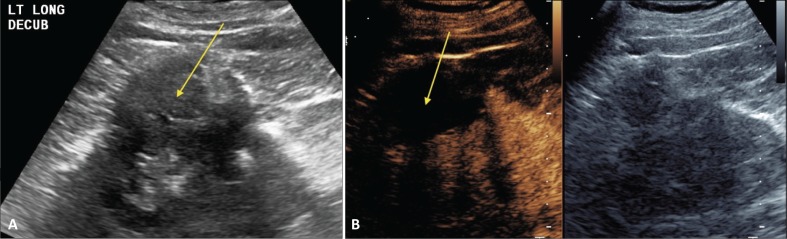
**Successful ablation.** Patient presented for 3-month post-RFA evaluation. (A) B-mode image before the ablation. Large heterogeneous tumor is noted on gray scale examination (arrow). (B) Absence of residual blood flow (arrow) is demonstrated on CEUS examination.

**Figure 4.**
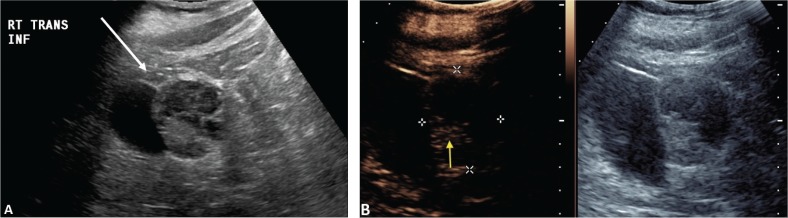
**Tumor recurrence/inadequate ablation.** Patient presented for CEUS examination 6 weeks after RFA. (A) The B-mode image demonstrates a complex mass (arrow) with solid and cystic components. It is not possible to determine if there is residual tumor. (B) CEUS image demonstrates the tumor (calipers) with residual flow in the posterior part of the tumor (arrow).

CEUS can be used to follow patients for residual or recurrent tumor with high accuracy. Any residual tumor will be identified as areas of increased enhancement. Absence of blood flow as in Figure 3B confirms complete ablation of the renal tumor. Note that it is not possible to determine if contrast is not utilized in Figure 3A. Figure 4 demonstrates a case where residual tumor (calipers) is present.

**Figure 5.**
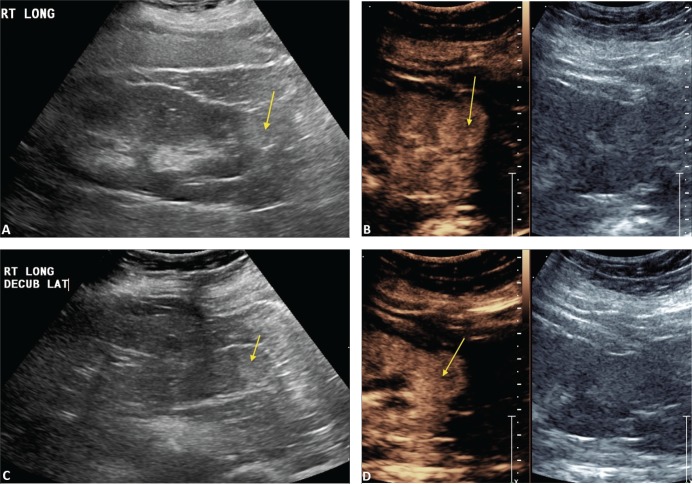
**Failed ablation.** Patient has history of prior left nephrectomy for RCC and presented with right renal mass. (A) Gray scale image of mass (arrow) pre-ablation. (B) CEUS demonstrating increased flow in tumor (arrow) at inferior pole of right kidney. (C) Gray scale image post-RFA of the mass (arrow). (D) CEUS post-RFA demonstrating flow throughout the tumor (arrow), indicating failed RFA.

Occasionally a complete ablation cannot be obtained. This is often from significant blood flow to the mass or the mass adjacent to the collection system which acts as heat sinks and does not allow for adequate temperature to ablate the tumor. Figure 5 demonstrates a renal mass on the B-mode image (A) and the CEUS image (B). Immediately post-RFA, the B-mode (C) and CEUS (D) demonstrate minimal ablation of the tumor.

**Figure 6.**
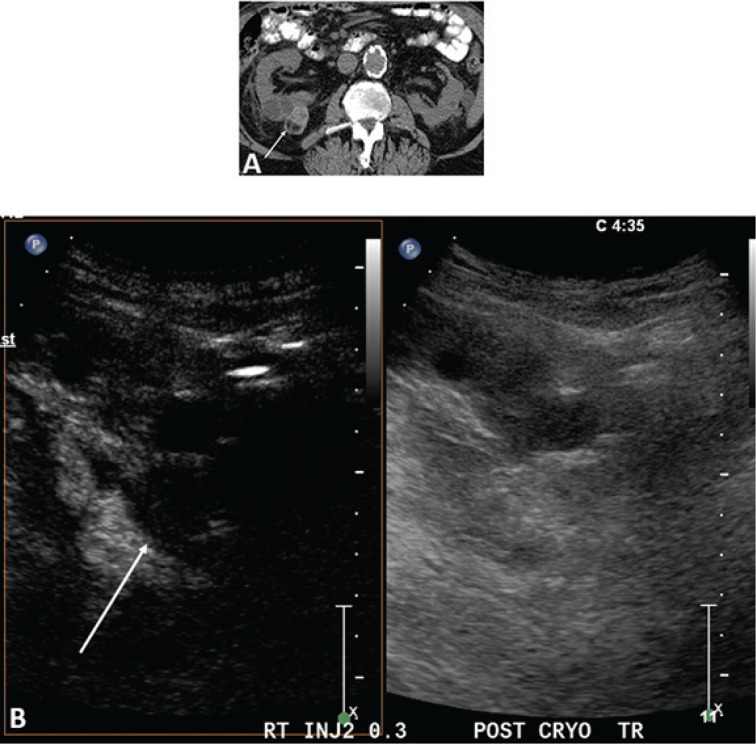
**Images 6 months post-RFA of an RCC.** (A) CT scan without contrast in this patient with renal failure. The ablated RCC (arrows) has significant attenuation making it difficult to determine if there is residual tumor. Even if contrast is administered, the background tissue makes it difficult to identify small areas of enhancement. (B) CEUS scan at the same 6-month post-RFA time point as the CT in (A). Ultrasound contrast agents can be used in patients with renal failure. Note that the excellent tissue suppression of CEUS allows for a contrast-only image, making it easy to see that there is no enhancement and no residual or recurrent tumor.

Figure 6 demonstrates the advantage of CEUS over CT. Because CEUS has a contrast-only image, small amounts of enhancement are easily identified. On CT (as well as MRI) there is signal from the associated soft tissues, making it difficult to identify small amounts of enhancement.

## Conclusion

Lately, there has been an increase in the use of CEUS to guide RFA of renal tumors. Using CEUS instead of CT as guidance has the advantages of providing a real-time image, being safe in patients with chronic renal disease, and being able to provide an immediate post-ablation image. When performing follow-up imaging to screen for residual tumor, CEUS yielded results similar to that of CT and MRI and has the advantage of a contrast-only image allowing for visualization of small amounts of enhancement. If studies continue to look favorably on CEUS as an alternative for evaluating and guiding the treatment of renal tumors, it may become an FDA-approved contrast method for RFA of renal tumors and provide a unique alternative to other imaging modalities.

## Conflict of Interest

R.G.B. has received research grants from Siemens Ultrasound, Philips Ultrasound, SuperSonic Imagine, GE Ultrasound, GE Medical, Bracco Diagnostics, and Lantheus Medical. He is on the Speaker’s Bureau of Philips Ultrasound, Bracco Diagnostics, and Lantheus Medical. He receives royalties from Thieme Publishers. C.P. is on the Speaker’s Bureau of Bracco Diagnostics. D.O. and T.C. declare no potential conflicts of interest with respect to research, authorship, and/or publication of this article.
